# Re: Angelo Cormio, Mario Auciello, Ugo Giovanni Falagario, et al. Mini and Standard Percutaneous Nephrolithotomy in Obese Patients. Results from a Single-surgeon Large Series. Eur Urol Open Sci 2024;63:113–8

**DOI:** 10.1016/j.euros.2024.05.007

**Published:** 2024-06-11

**Authors:** Wenjian Li

**Affiliations:** Department of Urology, Changzhou Third People’s Hospital, Changzhou Medical Center, Nanjing Medical University, Changzhou, China

I read the paper by Cormio et al. [1] with great interest and appreciate their efforts. The study offers insight into the effectiveness of mini- and standard nephrolithotomy (PCNL) in obese patients. The results demonstrate that mini-PCNL is associated with shorter surgical times, higher stone-free rates (SFRs), and lower blood transfusion rates in obese patients. These findings provide a new perspective on the treatment strategy for obese patients with renal stones and offer a valuable reference for related research.

However, while recognizing the academic value of the study, its conclusions need more evidence. The article states that mini-PCNL may be a better choice for obese patients because of its advantages over standard PCNL in terms of shorter surgical time, higher SFR, and lower transfusion rate. It is worth noting that the lower transfusion rate is generally recognized in the academic community. However, the results for comparisons of the surgical time and SFR appear to contradict the conclusions from most of the previous studies. Several meta-analyses have compared standard PCNL to mini-PCNL for the treatment of renal stones, and the results showed no significant difference in SFR between the two procedures, although surgical time is usually longer with mini-PCNL [2], [3], [4]. Therefore, a more comprehensive study is necessary to further confirm the authors’ results.

There is also room for improvement in the study design and implementation. Specifically, as a retrospective study, the data collection may have been affected by selection bias and incomplete information, which could have weakened the accuracy and reliability of the findings. To improve the scientific validity and persuasiveness of the study, future research should consider a multicenter prospective design to increase sample diversity and representativeness.

In addition, the study primarily focused on obese patients and did not fully consider other obesity-related parameters, which may impact the reliability of the findings. According to the authors, one study found that abdominal circumference, rather than body mass index or visceral fat area, was the primary factor affecting the effectiveness of PCNL. Therefore, future studies should include a broader range of anthropometric parameters for a more comprehensive evaluation of the impact of obesity on PCNL efficacy.

Finally, the authors controlled for a variety of potential confounders. However, several factors can still affect surgical outcomes and complications, such as the type of surgery, an intercostal approach, the degree of hydronephrosis, the number of approaches, and preoperative and postoperative hemoglobin differences [5]. To minimize the impact of confounding factors, the authors could use advanced statistical methods such as propensity score matching or instrumental variable analysis. In addition, long-term follow-up studies would help in comprehensive assessment of the long-term efficacy and safety of different PCNL approaches in obese patients.

This study provides valuable insights into the treatment of renal stones in obese patients. However, further research is necessary to improve many aspects of the study. I look forward to more high-quality research in the future, which will provide a more solid theoretical basis for clinical practice in this field.

  ***Conflicts of interest:*** The author has nothing to disclose.
